# Changes in the Difficulty of Endodontic Cases Treated by Undergraduate Students at a University Clinic Between 1971 and 2019

**DOI:** 10.1002/cre2.70071

**Published:** 2025-01-13

**Authors:** Pia Titterud Sunde, Erling Skallevold, Manpreet Kaur, Dag Solmund Ørstavik

**Affiliations:** ^1^ Department of Endodontics, Faculty of Dentistry University of Oslo Norway

**Keywords:** apical periodontitis, dental education, endodontic treatment, endodontics, molars

## Abstract

**Objective:**

This study aimed to assess the proportions of complicated endodontic cases treated by undergraduate dental students in a University clinic now and in the past.

**Material and Methods:**

Data were obtained from the electronic records and previous publications from the Faculty of Dentistry, University of Oslo, Norway. The operators were dental undergraduate students in their final 2 years of training. Proportions of tooth types, diagnosis, and retreatments were studied.

**Results:**

The amount of endodontically treated molars increased from 18% in 1971% to 44% in 2019. The number of teeth with preoperative apical periodontitis increased from 31% to 46%, and retreatments from 9% to 15%. The changes were highly significant, with the greatest change occurring from 1984 to 2009.

**Conclusion:**

Undergraduate students are exposed to more complicated teeth now than 50 years ago.

## 1 Introduction

Epidemiological studies show that dentists now treat more molars and teeth with AP than previously. Kirkevang et al. compared two Danish subpopulations (1974/1975 vs. 1997/1998) and found more endodontic treatment of molars in the later study sample (Kirkevang et al. 2001a). The authors speculated that there had been a change in the treatment strategy for teeth with AP; dentists try to treat and retreat and hesitate to extract teeth with periapical lesions.

In Denmark, it has been reported that most endodontic treatments are performed on teeth with caries and irreversible pulpitis. Even though AP was frequently noted in root‐filled teeth, retreatments were rare (2%). An increase in root‐filled teeth (20%) and root canals (36%) was found from 1977 to 2003 despite a reduction in caries among adults in Denmark. The authors speculated that the increased amount of root‐filled canals in their study was most likely due to more treatment of multiple rooted teeth and less extraction of teeth than in the past (Bjørndal and Reit 2004; Bjørndal, Laustsen, and Reit 2006).

Previous investigations in Jönköping, Sweden, also found an increased frequency of root‐filled molars over time, from 17% of all root‐filled teeth in 1973 to 33% in 2003 and 41% in 2013 (Frisk, Hugoson, and Hakeberg 2008; Silnovic, Kvist, and Frisk 2023; Hugoson et al. 1995).

It is well documented that endodontic treatment of teeth performed at dental educational institutions has a good prognosis (Ricucci et al. 2011; Sjögren et al. 1990; Ng, Mann, and Gulabivala 2011; Kerekes and Tronstad 1979; Llena et al. 2020). However, a lack of clinical experience in performing root canal treatments during undergraduate dental education is considered a problem. This may add to the various challenges of adapting into general practice; (Dahlström et al. 2017; Davey, Bryant, and Dummer 2015), and epidemiological studies report a high prevalence of AP in endodontically treated teeth of 40%–60% (Ridell et al. 2006; Kirkevang et al. 2001b; Skudutyte‐Rysstad and Eriksen 2006; Fransson et al. 2016; Koch et al. 2015).

Endodontic treatment is technically challenging and stressful for dental students. Low confidence in diagnosing endodontic disease and treating multi‐rooted teeth were the most frequently reported reasons for such a perception (Rolland, Hobson, and Hanwell 2007; Honey et al. 2011; Holmes, Diaz‐Arnold, and Williams 1997; Patel et al. 2006). The difficulty of localizing canals, complicated morphology, challenging accessibility (Mirza 2015), and difficulty achieving adequate anesthesia in the lower molar region are notable challenges for the dental student (Bigby et al. 2007; Claffey et al. 2004; Cohen, Cha, and Spångberg 1993).

Since 1969, undergraduate students at the Faculty of Dentistry in Oslo, Norway are required to treat a minimum of 15 root canals or 9 teeth (including 3 molars and one retreatment) in the endodontic curriculum.

Since epidemiological studies show a trend toward more treatment of molars, retreatments, and teeth with AP, it was desirable to investigate whether there is a similar trend also for undergraduate students at the Faculty of Dentistry in Oslo.

Therefore, the present study aimed to investigate the proportion of molars, retreatments, and teeth with AP treated at the Faculty of Dentistry, University of Oslo, Norway between 1971 and 2019.

The null hypothesis was that the proportion of molars, retreatments, and teeth treated with AP between 1971 and 2019 did not change significantly over time.

## 2 Materials and Methods

The study is part of a project reviewed and ethically approved by the Regional Ethics Board (REC south‐east) in Norway (64996 Resultatanalyse av endodontisk behandling).

Informed consent was not required since it was a quality control study looking at deidentified radiographs. The study included all patients treated with conventional endodontic treatment by undergraduate students and postgraduate students in endodontics at the department during the following intervals:

− August 1970 through June 1971 (data calculated from (Kerekes and Tronstad 1979))
− September 1981 through June 1982 [raw data retrieved from Ørstavik, Kerekes, and Eriksen (1987)]
− January through December 2005 [data retrieved from file collected from Marthinsen, Bones, and Ørstavik (2010)]
− January through December 2009 (data collected from the electronic records)
− January through December 2019 (data collected from the electronic records)

The patients treated in 1970–1971 and in 1981–1982 were treated exclusively by undergraduate students. The patients treated in 2005 were treated mainly by undergraduate but also by postgraduate students; however, the records did not permit a distinction by operator category. The patients treated in 2009 and 2019 were separated into groups treated by either under or postgraduates. In all, 9 groups of patients were defined and used in the analyses (Table 1).

**Table 1:** Patient groups by treatment provider.

Group	Provider
1971u	Undergraduates, August 1970 through June 1971
1982u	Undergraduates, September 1981 through June 1982
2009u	Undergraduates, January through December 2009
2019u	Undergraduates, January through December 2019
2009p	Postgraduates, January through December 2009
2019p	Postgraduates, January through December 2019
2005a	Under‐ and postgraduates, January through December 2005
2009a	Under‐ and postgraduates, January through December 2009
2019a	Under‐ and postgraduates, January through December 2019

Abbreviations: a, all cases; p, postgraduates; u, undergraduates.

Data of tooth type (front teeth, premolars, molars), type of treatment performed (pulp extirpation, primary treatment of AP, retreatments), and periapical status (presence or absence of apical periodontitis) were registered for all patients.

Data for the presence or absence of apical periodontitis could not be extracted for the group of patients treated in 2005; thus, this data set was not used for comparisons of preoperative periapical diagnosis. The raw data in Kerekes and Tronstad (Kerekes and Tronstad 1979) did not specify diagnosis and treatment per tooth but per root; therefore, we estimated these parameters by calculations based on the information in their Table 1 (Kerekes and Tronstad 1979). The periapical status in this 1970–1971 patient group was recorded as radiographically normal, uncertain, or apparent radiolucency, whereas the 1982, 2005, 2009, and 2019 data used the Periapical Index (PAI) scored by observers calibrated in PAI using a set of reference radiographs of teeth with scores qualified with expert consensus. PAI scores 3–5 indicate the presence of apical periodontitis (Orstavik, Kerekes, and Eriksen 1986). A PAI score of 3 would represent “uncertain” in the 1970–1971 group of patients; therefore, “uncertain” was pooled with “apparent radiolucencies” to represent apical periodontitis in that patient group.

### 2.1 Statistical Methods

Distributions of categories of tooth and treatment types and preoperative diagnosis in the selected groups of patients were compared by chi‐square tests.

The chi‐square test was used also when several groups within the same category were compared. The level of significance was set to *p* < 0.05.

## 3 Results

The distributions of the recorded parameters are all given in Table 2.

**Table 2:** Distributions of treatment types, tooth types, and periapical diagnoses.

Tooth characteristic	1971u	1984u	2009u	2019u	2009p	2019p	2005a	2009a	2019a
Front teeth	203	221	132	91	40	58	114	172	149
Premolars	187	183	179	148	39	40	121	218	188
Molars	88	169	208	191	104	110	186	312	301
Pulp extirpation	242	255	226	147	44	26	133	270	173
Primary AP	194	242	211	220	67	112	199	278	332
Retreatment	42	76	82	63	72	70	89	154	133
Normal apical periodontium	308	387	307	230	73	82		380	312
Apical periodontitis	170	186	212	200	110	126		322	326
All	478	573	519	430	183	208	421	702	638

Abbreviations: a, all cases; p, postgraduates; u, undergraduates.

### 3.1 Changes in Tooth Type

**Figure 1:**
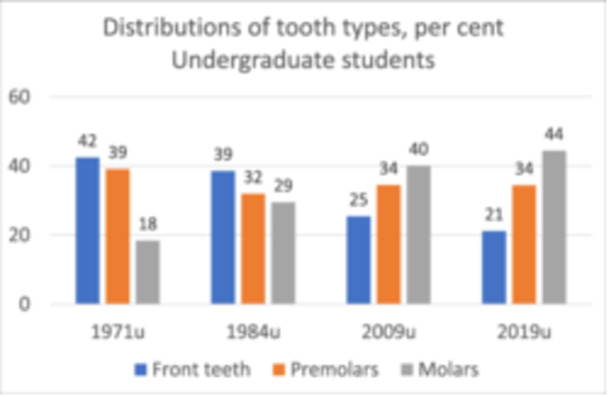
Changes in the distribution of tooth types from 1971 to 2019 shown as percentages. Teeth treated by undergraduate students. *p* = 4.43E‐21 by chi‐square.

There was a marked shift in the tooth type treated by undergraduate students over the decades studied. Front teeth constituted nearly half the caseload in 1971, gradually decreasing to less than a quarter by 2019. Premolars were also reduced in proportion from 1971 to 1984, then stabilized around 32%–34%. The proportion of molars more than doubled from 18% in 1971% to 44% in 2019 (Figure 1). The trend was significant (*p* < 0.01) from 1971 to 1982 and 2009 but not significant from 2009 to 2019.

Relative to the cases treated by undergraduate students, the caseload for postgraduate students was shifted even more toward more treatment of molars (Table 2).

### 3.2 Changes in the Type of Endodontic Treatment Provided

**Figure 2:**
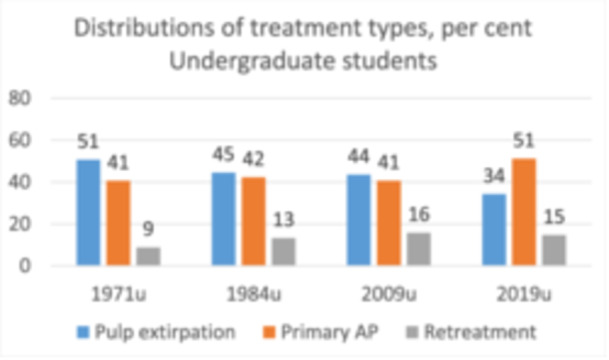
Changes in the distribution of treatment types from 1971 to 2019 are shown as percentages. Teeth treated by undergraduate students. *p* = 1.22E‐05 by chi‐square.

Retreatments performed by undergraduates were 9% in 1971% and 15% in 2019. Pulp extirpation declined steadily from 51% to 34%, whereas treatment of primary apical periodontitis increased particularly from 2009 to 2019 (*p* < 0.01) (Figure 2).

### 3.3 Changes in Preoperative Periapical Diagnosis

**Figure 3:**
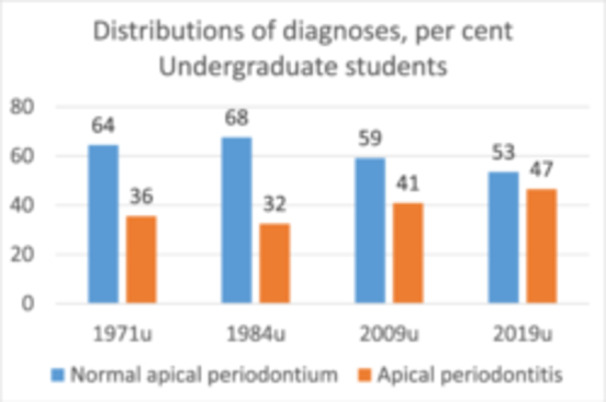
Changes in the distribution of periapical diagnosis from 1971 to 2019 are shown as percentages. Teeth treated by undergraduate students. *p* = 3.26E‐05 by chi‐square.

The relative number of teeth with preoperative apical periodontitis treated by undergraduates increased from 36% in 1971 to 47% in 2019 (Figure 3). The trend was significant only from 1984 to 2009 (*p* < 0.01).

Graduate and undergraduate cases could be pooled for the years 2005, 2009, and 2019. There was no significant change in any distribution for all cases treated in the department (2005a, 2009a, and 2019a) nor for cases treated by postgraduate students (2009p and 2019p) (Table 2).

## 4 Discussion

The present study retrospectively compared the type of tooth, treatment type, and the number of teeth with preoperative AP subjected to root‐filling at the Faculty of Dentistry from 1971 until 2019. The results showed that there had been significant changes in the caseload for undergraduate students.

The proportion of endodontically treated molars treated by undergraduate students increased from 18% in 1971 to 44% in 2019. The number of teeth with AP increased from 36% in 1971 to 47% in 2019. As much as 62% of the teeth treated by postgraduate students in 2019 had preoperative apical periodontitis.

Variation in how data was obtained over time is a study limitation. Data were collected from electronic records or published papers, and the quality of the data registered could not be ascertained. However, the tooth type data seem solid, and the assumptions made regarding diagnosis and type of treatment should reflect relevant clinical practices. Data on the presence of apical periodontitis could not be extracted from the 2005 group, so this year was omitted from the assessment of undergraduate students' cases.

Several factors may contribute to variations in case complexity, e.g., canal obliteration, age, and other tooth‐ and patient‐associated factors. We focused on the treatment of molars, retreatments, and treatment of apical periodontitis as an important and easily recordable measure for complex cases.

Studies from Dental schools looking at endodontic treatment by undergraduate students report conflicting results. Comparisons are difficult due to differences in study design and the fact that different operators perform treatments: students, post‐graduates, or staff. Root canal treatment performed by dental students at the University of Pennsylvania between 1947 and 1952 showed that 90% were front teeth and premolars, and only 10% were molars. Twenty‐three per cent of the teeth had preoperative apical periodontitis (Grossman, Shepard, and Pearson 1964). Similarly, Molven et al. found that of root canal treatments performed by undergraduate students at the Faculty of Dentistry in Bergen, Norway, during 1963–1969, approximately 15% were molars, and 25% had apical periodontitis (Molven 1976; Molven and Halse 1988). On the other hand, of endodontic treatments carried out by undergraduate students at the Department of Endodontics, Faculty of Dentistry in Helsinki from 1964 to 69, 30% had AP before treatment, and as much as 56% were molars (Jokinen et al. 1978).

Similar studies from general dental practice also show conflicting results. Of the root fillings performed from 1968 to 1977 in private dental practice in the USA, 32% were molars, and only 4% had preoperative apical periodontitis (Barbakow, Cleaton‐Jones, and Friedman 1980). Another study from a private practice in Germany found that 47% of the root‐filled teeth in 2001 were molars (Skupien et al. 2013). Of 420 endodontic referrals treated between 1996 and 2004 in the Endodontic Clinic at the Public Dental Health, Gothenburg, Sweden, 37% were molars, and as much as 86% had apical periodontitis (Landys Borén, Jonasson, and Kvist 2015). Another study from Sweden found that of 243 teeth undergoing root canal treatment in 2015–2016 in a public dental service, 48% were molars, and 38% had preoperative apical periodontitis (Wigsten, Jonasson, and Kvist 2019).

Our study found that dental students at the Faculty of Dentistry treat more molars than in the past. There also seems to be a trend in the general population that more molars are endodontically treated today than earlier.

Dental health among 35‐year‐olds in Oslo, measured as a reduction in caries experience, improved considerably from 1973 to 2003 (Skudutyte‐Rysstad and Eriksen 2007). However, the proportion of root‐filled teeth with apical periodontitis increased from 18% in 1973% to 43% in 2003 despite the improved technical quality of root fillings (Skudutyte‐Rysstad and Eriksen 2006). In a recent study from Oslo, Norway, looking at the endodontic status of 65‐year‐olds, it was found that 35% of all root‐filled teeth had apical periodontitis, and molars had the worst outcome (Diep et al. 2022).

Studies from Sweden also report on improved technical quality of root fillings from 1973 to 2003, without an improvement of the periapical status in root‐filled teeth (Frisk, Hugoson, and Hakeberg 2008). The authors assumed that the larger proportion of molars in 2003 contributed to the result (17.3% in 1973 vs. 33.4% in 2003). A later cross‐sectional study from Sweden by the same group showed that 41% of all root‐filled teeth in 2013 were molars and had more often apical periodontitis (46%) than premolars (22.4%) and incisors (29.3%) (Silnovic, Kvist, and Frisk 2023) The same results were found in a cross‐sectional study by Kirkevang et al. in 2017: the apical status of endodontically treated teeth differed considerably amongst tooth groups, with molars being at higher risk of a poorer outcome than more anterior teeth (Kirkevang et al. 2017). Laukkanen et al (Laukkanen, Vehkalahti, and Kotiranta 2021). reported on the outcome of root canal treatment in general dental practice. They found that molars had the worst outcome compared to incisors and premolars, both regarding the healing of AP and the technical quality of the root canal filling.

The findings reported above likely reflect that patients wish to retain their teeth rather than extract them, but they also point out the technically difficult challenge of performing root canal treatment on molars. When dental students treat more molars and perform more retreatments today than earlier, they treat more technically complicated cases than before. This probably leads to more frequent complications during treatment. One study used the American Association of Endodontics (AAE) Case Difficulty Assessment Form to grade levels of difficulty and assess the occurrence of endodontic mishaps in an undergraduate student clinic. They found molars significantly more often in the moderate‐ to high‐difficulty category. The moderate‐ to high‐difficulty cases experienced more mishaps (65%) than minimal‐difficulty cases (35%) (Almohaimede et al. 2022).

Fifth‐year graduate dental students in the School of Dentistry, University of Jordan, reported a lack of confidence in complex procedures that were the least practiced, such as root canal treatment of posterior teeth (Hattar et al. 2021). The self‐efficacy and competence of students were influenced only by the number of tutorials and the quantity of root canal treatments performed under the supervision of endodontists (Baaij and Özok 2018). Moreover, the same authors found that students' self‐efficacy was primarily influenced by how much clinical experience they had when performing root canal treatment (Baaij et al. 2020). The more root canal treatments students had performed on patients, the greater their self‐efficacy at graduation. However, they also found that treating complex cases (molars and retreatments) might reduce their self‐efficacy. A study that evaluated dental students' anxiety related to endodontic treatment showed that their anxiety diminished as they gained more experience in endodontic procedures (Luz et al. 2019). In addition, self‐efficacy increased if root canal treatments were performed within the first year following graduation (Baaij et al. 2021).

Looking at the relatively poor outcome of endodontic treatment in epidemiological studies worldwide in comparison to treatment performed in dental institutions, effort should be made to train undergraduate students to perform root canal treatments while they are students and receptive to information. Training in endodontics for dental students is crucial for establishing good technical skills and treatment routines. To succeed as competent future dentists, students must treat a sufficient amount of molars, and the curriculum and passing requirements should be adapted accordingly.

## 5 Conclusions

The present study showed that an increased number of molars, retreatments, and teeth with AP are being treated now than in the past by undergraduate students at the Department of Endodontics, Faculty of Dentistry in Oslo, Norway.

## Author Contributions

Pia Titterud Sunde drafted the article, contributed to the conception, and design of the study. Dag Ørstavik contributed to the conception and design of the study, interpretation of data, and revising the article critically. Erling Skallevold collected data and contributed to the concept of the article. Manpreet Kaur collected data and contributed to the concept of the article.

## Ethics Statement

The study is part of a project reviewed and accepted by the Regional Ethics Board (REC south‐east) in Norway (64996 Resultatanalyse av endodontisk behandling).

## Conflicts of Interest

The authors declare no conflicts of interest.

## Data Availability

The data may be available upon appropriate request.
